# Hibernating bear serum triggers an anti-fibrotic signature in human fibroblasts, involving ECM remodeling and MAPK signaling activation

**DOI:** 10.1038/s41598-026-43734-y

**Published:** 2026-03-21

**Authors:** Jade Sutter, Alexandre Geffroy, Amandine Moretton, Anne Randi Græsli, Jonas Kindberg, Lydie Combaret, Etienne Lefai, Isabelle Garreau-Balandier, Fabrice Bertile, Patrick Vernet

**Affiliations:** 1 Université Clermont Auvergne, CNRS, LPCA, 63000 Clermont-Ferrand, France; 2 Université de Strasbourg, CNRS, IPHC UMR7178, Strasbourg, France; 3 Proteomics French Infrastructure, PROFI-CORE UAR2048, Strasbourg, France; 4 Department of Forestry and Wildlife Management, Faculty of Applied Ecology and Biotechnology, University of Inland Norway, Campus Evenstad, Koppang, Norway; 5 Norwegian Institute for Nature Research, Trondheim, Norway; 6 Department of Wildlife, Fish and Environmental Studies, Swedish University of Agricultural Sciences, Umeå, Sweden; 7 Université Clermont Auvergne, INRAE, UNH UMR 1019, CRNH Auvergne, Clermont- Ferrand, France

**Keywords:** Extracellular matrix, Fibrosis, Phosphorylation, Signaling, Fibroblast, Biochemistry, Biomarkers, Cell biology, Diseases, Molecular biology, Physiology

## Abstract

Some hibernating species, such as the brown bear (*Ursus arctos*), can endure several months of physical inactivity and fasting without suffering the typical deterioration observed in inactive or starved non-hibernators, such as muscle atrophy, bone loss or systemic inflammation. The underlying physiological and molecular adaptive mechanisms may involve humoral factors circulating in the blood, which could help maintain cellular and tissue homeostasis under prolonged stress. To investigate the intracellular mechanisms triggered by blood components from hibernating bears, we treated human fibroblast cells with serum from either active bears, hibernating bears, or fetal bovine serum as controls. We then performed an untargeted proteomics analysis to identify changes in the treated cells. This revealed remodeling of extracellular matrix (ECM) and modulation of key regulatory pathways, indicating an anti-fibrotic signature in cells treated with hibernating bear serum. In particular, it was characterized by reduced levels of fibrotic markers such as collagens (COL2A1, COL11A1, COL9A1) and the TGF-β1-associated signaling component TGFBI, as well as activation of the MAPK cascade. Additionally, a decrease in the levels of inflammatory markers (IFI16, HMGB2, and HMGB), which play a key role in the fibrosis process, was observed. Overall, our study provides new evidence that hibernation-related circulating factors are associated with changes in pro-fibrosis pathways and ECM homeostasis in human cells. These findings suggest that hibernating bear serum may serve as a valuable model to explore mechanisms underlying anti-fibrotic gene expression programs and to identify potential targets for tissue preservation.

**Supplementary Information:**

The online version contains supplementary material available at 10.1038/s41598-026-43734-y.

## Introduction

In mammals, homeostasis relies on a dynamic balance between the synthesis and degradation of cellular constituents, a balance that is highly dependent on environmental and metabolic factors, particularly nutritional status and level of physical activity. Disruption of this balance, for instance in the case of prolonged fasting and immobilization, leads to a series of well-documented pathophysiological responses, including muscle wasting, bone demineralization, oxidative stress and onset of chronic systemic inflammation^1,2^. In contrast, some hibernating animals, such as the brown bear (*Ursus arctos*), experience prolonged periods of physical inactivity and total fasting without suffering significant tissue damage^3^. During hibernation, which can last several months, these animals retain their muscle mass and bone density, and maintain minimal but sufficient metabolic activity to ensure homeostasis^4,5^. Powerful endogenous protective mechanisms capable of preserving tissues despite extreme conditions must therefore be at work.

Several studies have shown clear effects of hibernator serum on various cultured cell types, suggesting that circulating signals likely mediate the cellular adaptations associated with hibernation^6–11^. In particular, bear serum collected during hibernation contains a wide variety of bioactive molecules (i.e. hormones, cytokines, growth factors, microRNAs, and regulatory proteins) that could contribute to cellular protection and the maintenance of tissue homeostasis^12–14^. When used as a supplement in cell culture media in vitro, this winter serum induces cytoprotective effects^7,8^ and a hypertrophic response with inhibited proteolysis reported in muscle cells^6^. More specifically, some pathways modulated in muscle cells exposed to hibernating bear serum, such as the TGF-β and AKT pathways, have been suggested to contribute to the increase in their protein content^6,15,9^. However, a detailed analysis of how other intracellular processes and tissue homeostasis are affected by circulating signals from bear serum remains to be performed.

In this study, we investigated the effects of serum from active or hibernating bears (summer vs. winter) compared to serum from non-hibernating animals (bovine) on human fibroblasts in culture using large-scale proteomics analysis. Fibroblasts were chosen because they are robust, easy to culture, and sensitive to soluble factors in serum, making them a suitable model to study serum-mediated effects on cells^16^. Our results revealed a molecular signature suggestive of an anti-fibrotic effect in cells treated with winter bear serum, with a decrease in the abundance of extracellular matrix (ECM) components and in immune related molecules, along with activation of the MAPK cascade. Overall, our findings demonstrate that hibernating bear serum treatment is associated with changes in cellular processes related to tissue remodeling and preservation. These results highlight the potential of hibernating bear serum as a tool for studying natural mechanisms of tissue protection, and may help identify candidate molecular regulators of fibrosis and extracellular matrix homeostasis.

## Materials and methods

### Brown bear blood sample collection and serum mixes preparation

Capture, anesthesia and sampling were performed as previously described^17,18^. In summer, all bears were darted from a helicopter with a remote drug delivery system (DANiNJECT^®^) with a combination of medetomidine (M) and tiletamine-zolazepam (TZ). For small bears such as in our study (2–3 years, 45–70 kg), the intramuscular injection of a fixed M: TZ ratio is used (2.5 mg M + 125 mg TZ). In winter, bears are darted while still in their den. For 2 y-old individuals (30–45 kg), the intramuscular injection of a fixed M: TZ ratio is used (0.5 mg M + 62.5 mg TZ) and ketamine (K) is added (50 mg). For 3 y-old individuals (45–60 kg), doses are doubled (1 mg M + 125 mg TZ + 100 mg K). During anesthesia, all bears were administered supplemental oxygen; ears and eyes were covered with a blindfold; and eye gel was administered to the cornea to avoid drying. To reverse the effects of medetomidine, atipamezole at 5 mg per mg of total medetomidine dose was administered intramuscularly and the bears were released in quiet areas so that they could recover safely (in their dens in winter; in the shade and away from water and main roads in summer).

Nineteen free-ranging brown bears (4 males and 15 females aged 2–3 years) were captured during their hibernation (February) and active period (June) in Dalarna and Gävleborg counties in Sweden between 2015 and 2020. Blood samples were taken from the jugular vein within 20 min after darting using tubes containing a clotting activator (Vacuette Z Serum Sep Clot Activator, Greiner Bio-One GmbH, Kremsmünster, Austria). Centrifugation (3000 x g, 20 min) was then performed within 1 h after sampling and serum was immediately frozen on dry ice until storage at −80 °C. Equal quantities of serum from the winter collection on the one hand and the summer collection on the other hand were mixed to obtain a winter bear serum pool (WBS) and a summer bear serum pool (SBS), respectively.

All animal experiments reported in this manuscript were conducted in compliance with the ARRIVE guidelines^19^ and all procedures were approved by the Swedish Ethical Committee on Animal Experimentation (Dnr C 286/12 and C 3/2016), the Swedish Environmental Protection Agency (NV-01758–14 and NV-00741–18), and the Swedish Board of Agriculture (Dnr 5.2.18–3060/17). All procedures complied with Swedish laws and regulations.

## Human fibroblasts HFF-1 treatment with FBS, SBS and WBS

Human foreskin fibroblasts HFF-1 (SCRC-1041; ATCC) were seeded into 6-well plates and allowed to adhere for 24 h in standard DMEM medium containing 10% fetal bovine serum (Biowest), at 37 °C and 5% CO_2_.

**Fig. 1:**
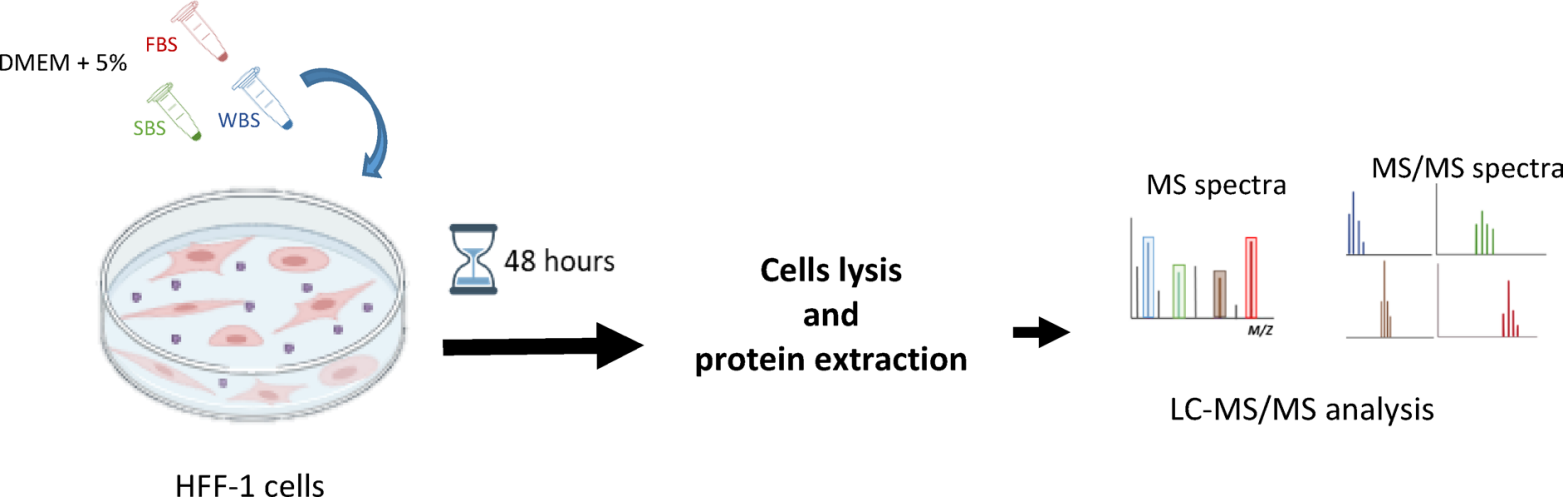
Schematic representation of the experimental protocol.

For treatment with bear serum, medium was removed and replaced with DMEM containing either 5% Winter Bear Serum (WBS), 5% Summer Bear Serum (SBS), or 5% Fetal Bovine Serum (FBS) as control condition. Cells were then incubated at 37° for 48 h, then lysed for total protein extraction. Three replicates were considered for SBS and FBS and four for WBS conditions (Fig. 1).

## Sample preparation for proteomic analysis

Sample preparation, nanoLC-MS/MS analysis, and mass spectrometry data processing (see below) were performed according to a previously published protocol^20^, which we have modified below to reflect all changes we made to adapt it to HFF1 samples.

After treatment, cells were directly lysed in each well using Laemmli sample buffer (0.06 M Tris-HCl pH 6.8, 0.2% bromophenol blue, 10% glycerol, 2% SDS, 5% beta-mercaptoethanol). Lysates were collected and stored at −80 °C until analysis. Total protein concentration was determined using the RC-DC Protein Assay kit following manufacturer’s instructions (Bio-Rad, Hercules, CA, USA). At this stage, a reference sample comprising equal amounts of all protein extracts was made, to be analyzed regularly during the whole experiment and allow QC-related measurements. 20 µg of proteins from each sample were loaded onto SDS-PAGE gels (5% polyacrylamide for the stacking gel and 12% for the resolving gel) and electrophoresed for 32 min at 50 V then 16 min at 100 V. Proteins were thereafter fixed by a 20 min incubation of gels in a solution composed of 50% ethanol and 3% phosphoric acid. Staining was performed using colloidal Coomassie Blue (20 min), and visualization of proteins allowed six protein bands (2 mm each) to be excised from the resolving gel. The portion of the stacking gel was also excised to enable the analysis of any protein remaining there.

After destaining using 25 mM acetonitrile/ammonium hydrogen carbonate (50/50, v/v, 5 × 5 min at RT) and dehydration using pure acetonitrile (5 min at RT), proteins were reduced and alkylated in-gel using 10 mM DTT in 25 mM ammonium hydrogen carbonate buffer (30 min at 60 °C) and 55 mM iodoacetamide in 25 mM ammonium hydrogen carbonate buffer (20 min at RT in the dark), respectively. Gel slices were then washed using 25 mM ammonium hydrogen carbonate buffer (5 min at RT) and acetonitrile (5 min at RT) four times, and dehydration was finally performed using pure acetonitrile (2 × 5 min at RT). In-gel digestion of proteins was performed overnight at 37 °C using trypsin (Promega Madison, WI, USA; 100 ng per band), and the resulting peptides were extracted (1 h) on an orbital shaker (110 rpm) using a solution composed of 80% acetonitrile and 0.1% formic acid in water. Organic solvent was thereafter evaporated using a vacuum centrifuge (SpeedVac) and the volume of peptide extracts was adjusted to 25 µL using a solution composed of 2% acetonitrile and 0.1% formic acid in water. At this stage, a set of reference peptides (iRT kit; Biognosys AG, Schlieren, Switzerland) was added to peptide extracts (2.5 µL/sample after resuspension in 50 µL of the kit dissolution buffer) for QC-related measurements.

## nanoLC-MS/MS analysis

NanoLC-MS/MS analysis was performed using a nanoUPLC-system (nanoAcquity; Waters, Milford, MA, USA) coupled to a quadrupole-Orbitrap hybrid mass spectrometer (Q-Exactive HF-X; Thermo Scientific, San Jose, CA, USA). The system was fully controlled by XCalibur software (v4.0.27.19; ThermoFisher Scientific). Samples (1 µl) were first concentrated/desalted onto a NanoEAse M/Z Symmetry precolumn (C18, 100 Å, 5 μm, 180 μm × 20 mm; Waters) using 99% of solvent A (0.1% formic acid in water) and 1% of solvent B (0.1% formic acid in acetonitrile) at a flow rate of 5 µl/min for 3 min. A solvent gradient from 5 to 6% of B in 0.5 min then from 6 to 35% of B in 43 min was used for peptide elution, which was performed at a flow rate of 350 nL/min using a NanoEAse M/Z BEH column (C18, 130Å, 1.7 μm, 75 μm x 250 mm; Waters) maintained at 60 °C. Samples were analyzed randomly per block, each block being composed of one biological sample from each group. The reference sample was analyzed five times throughout the experiment. In between each sample, regeneration of the column using 50% acetonitrile during 17 min and running of a solvent blank allowed limiting carry-over effects. Peak intensities and retention times of reference peptides were monitored in a daily fashion.

The Q-Exactive HF-X was operated in positive ion mode with source temperature set to 250 °C and spray voltage to 2.1 kV. Full-scan MS spectra (150–2200 m/z) were acquired at a resolution of 120 000 at m/z 200. MS parameters were set as follows: maximum injection time of 50 ms, AGC target value of 1 × 10^6^ ions, lock-mass option enabled (polysiloxane, 445.12002 m/z), selection of up to 20 most intense precursor ions (doubly charged or more) per full scan for subsequent isolation using a 2 m/z window, fragmentation using higher energy collisional dissociation (HCD, normalized collision energy of 27), dynamic exclusion of already fragmented precursors set to 40 s. MS/MS spectra (dynamic first mass) were acquired with a resolution of 15,000 at m/z 200. MS/MS parameters were set as follows: maximum injection time of 60 ms, AGC target value of 1 × 10^5^ ions, peptide match selection option turned on.

## Mass spectrometry data processing

Raw data were processed using MaxQuant v2.6.3.0^21^. Peak lists were created using default parameters. Using Andromeda search engine implemented in MaxQuant, peaklists were searched against a Swiss-Prot protein database (Homo sapiens, TaxID 9606; 20418 entries) created in March 2025 with MSDA software suite^22^. The database was complemented by Andromeda with the addition of the sequences of common contaminants like keratins and trypsin (246 entries) and of decoy (reverted) sequences for all Homo sapiens proteins. Parameters were set as follows: precursor mass tolerance set to 20 ppm for the first search and to 4.5 ppm for the main search after recalibration, fragment ion mass tolerance set to 20 ppm, carbamidomethylation of cysteine residues considered as fixed modification, oxidation of methionines and acetylation of protein N-termini considered as variable modifications, peptide length of minimum 7 amino acids, maximum number of trypsin missed cleavages set to one, false discovery rate (FDR) set to 1% for both peptide spectrum matches and proteins. The proteins identified with less than two peptides or with a negative score were discarded from identification data, as well as decoy hits and potential contaminants.

Protein quantification was performed using the MaxLFQ (label-free quantification) option implemented in MaxQuant^21^. Parameters were set as follows: “minimal ratio count” of one, “match between runs” option enabled using a 0.7-minute time window after retention time alignment, consideration of both unmodified and modified (acetylation of protein N-termini and oxidation of methionines) peptides for quantitative determination, exclusion of shared peptides. All other MaxQuant parameters were set as default. Finally, criteria for retained proteins were as follows: at least one unique peptide quantified and at least three values per group. Proteins absent in given groups (i.e. not detected at all) but satisfying above-mentioned criteria for the other groups were also retained. The mass spectrometry proteomics data have been deposited to the ProteomeXchange (https://www.proteomexchange.org/) Consortium via the PRIDE^23^ partner repository with the dataset identifier PXD069199.

From QC-related measurements, we could see that the whole analysis system remained stable throughout the experiment. Indeed, a median coefficient of variation (CV) of 1.0% was calculated for retention times of iRT peptides over all injections, and a median CV of 12.2% was computed for all LFQ values obtained from the repeated analysis of the reference sample.

### Protein extraction and MAPK phosphorylation analysis

Phosphorylation levels of key components of the MAPK pathway were assessed using the Human/Mouse MAPK Phosphorylation Array C1 kit (RayBiotech, AAH-MAPK-1). According to the manufacturer’s instructions, proteins were extracted directly from the cell culture wells using the commercial lysis buffer from the kit. The kit consists in membranes pre-coated with the following antibodies: Akt/PKB (P-Ser473), CREB (P-Ser133), ERK1 (P-T202/Y204)/ERK2 (P-Y185/Y187), GSK3a (P-Ser21), GSK3b (P-Ser9), HSP27 (P-Ser82), JNK (P-Thr183), MEK (P-Ser217/Ser221), MKK3 (P-Ser189), MKK6 (P-Ser207), MSK2 (P-Ser360), mTOR (P-Ser2448), p38 (P-Thr180/Tyr182), P53 (P-Ser15), P70S6K (P-Thr421/Ser424), RSK1 (P-Ser380) and RSK2 (P-Ser386). One membrane was used per condition. Membranes were first blocked for 1 h, after which an equal amount of protein was loaded onto each membrane. They were then incubated overnight at 4 °C with the protein samples under gentle agitation. After incubation and subsequent washing steps, membranes were treated with a cocktail of biotin-conjugated detection antibodies allowing specific binding to the captured phosphorylated proteins. Subsequently, HRP-conjugated streptavidin was applied to recognize the biotin tags and enable enzymatic signal amplification. Chemiluminescent signals were then generated using an enhanced chemiluminescence (ECL) substrate and captured with a digital imaging system (Vilber). Spot intensities were quantified with ImageJ software, after background-subtraction and normalized to internal positive controls.

## Statistical analysis and functional bioinformatics analysis

For the proteomic data analysis, the R software environment (v4.2.2) was used to check for data normal distribution (Shapiro-Wilk;* p*-value > 0.01) and homoscedasticity (Bartlett;* p*-values > 0.01). The comparison of the proteome among the three groups (FBS, SBS and WBS) was performed using one-way ANOVA and Tukey tests with honest significant difference (HSD) correction for multiple comparisons (p-value < 0.05). Only proteins differentially expressed with a* p*-value < 0.05 were considered for bioinformatics analysis. Functional annotation enrichment analysis using Gene Ontology (GO) terms was performed using Metascape (www.metascape.org)^24^. Default statistical settings were used and GO terms with adjusted* p*-values < 0.05 were considered significantly enriched. Hierarchical clustering and visualization of the results as a heatmap was performed using R with the ComplexHeatmap package, within an R Markdown notebook environment. Prior to visualization, data were log-transformed and standardized (row-wise z-score). Clustering was performed using Pearson correlation for distance measurement and Ward method for linkage. Also, in R, a principal component analysis (PCA) was performed to evaluate the extent of variation in the proteomic data and was able to discriminate samples from FBS, SBS and WBS conditions. Protein intensities were centered and reduced, and submitted to the prcomp() function in R. The proportion of variance explained by each axis was extracted and visualized with fviz_eig() from the factoextra package. Protein contributions to the first two principal components (PC1 and PC2) were calculated from get_pca_var() and saved for the identification of discriminating variables. The projection of observations onto the factorial plane was performed and represented by ggplot2. Statistical analysis of the MAPK pathway was conducted using one-way ANOVA followed by Tukey tests.

## Results

### Serum treatment induces proteomic changes: fetal bovine vs. hibernating and active subadult bear serum effects

**Fig. 2:**
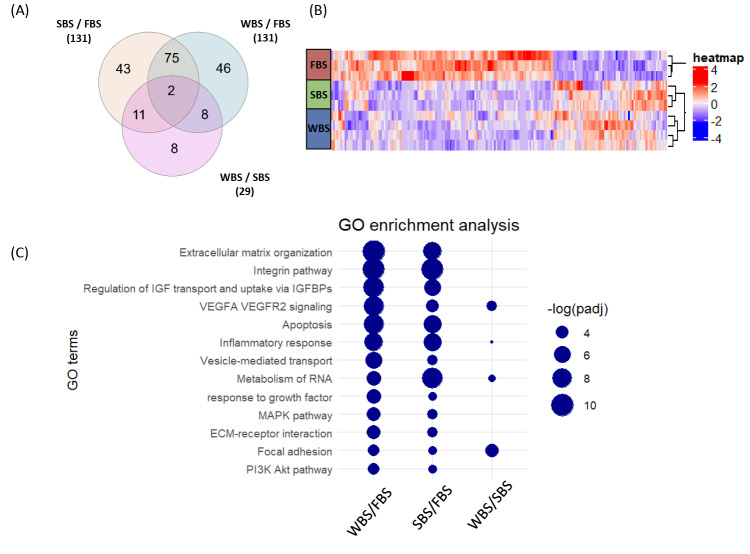
Differential protein expression and associated functional annotation enrichment analyses. (**A**) Venn diagram showing the distribution of the 193 differentially expressed proteins across the experimental conditions. (**B**) Hierarchical clustering displaying the relative abundance of proteins across conditions. (**C**) Dotplot of selected enriched terms as identified by Metascape analysis for the following pairwise comparisons: FBS vs. WBS, FBS vs. SBS and SBS vs. WBS.

Proteomics allowed 4738 proteins to be detected, including 2829 for which quantification could be retained (Table S1) according to our stringent filtering criteria (see Methods). In total, 193 proteins were found differentially expressed, and the distribution among the three experimental conditions are displayed in Fig. 2A. A marked effect between fetal bovine and subadult bear serum conditions was observed, with 131 proteins differentially expressed in SBS- vs. FBS-treated cells and in WBS- vs. FBS-treated cells. Twenty-nine proteins were also differentially expressed between the WBS and SBS conditions, indicating a slightly different effect of the winter versus the summer serum (Fig. 2A). Hierarchical clustering showed a clear discrimination of the biological replicates from different groups (Fig. 2B). This reflected low inter-replicate variability within a given condition and confirmed the reproducibility of quantitative data. The dendrogram also revealed a clear separation between the FBS condition and the two bear serum conditions, which cluster together. Despite that, patterns of differentially abundant proteins distinguish the SBS and WBS serum conditions to each other, clearly suggesting proteome changes following incubation with hibernating bear serum vs. active bear serum (Fig. 2B). This clear discrimination is moreover nicely seen using principal component analysis (Fig. S1A). The first two components of the PCA explain 53.1% and 15.4% of the total variance, respectively (Fig. S1B). The observed separation does not result from the contribution of a single major protein, but rather from the combined effect of a large set of proteins showing variation (Fig. S1C). This suggests that the differences between conditions are based on global protein signatures rather than a single or a small number of biomarkers.

### Functional annotation enrichment analysis reveals an impact of bear serum on matrix remodeling and cell-matrix environment

Functional annotation enrichment analysis of biological processes from Gene Ontology (GO) knowledgebase was performed using Metascape (metascape.org; Fig. S2). The main changes, which are summarized in Fig. 2C, highlight that several enriched GO terms are associated with extracellular matrix (ECM) organization, ECM-receptor interaction, and focal adhesion in SBS/FBS and WBS/FBS comparisons. Enrichment in inflammatory response and RNA metabolism GO terms is also to be noted. Given that clotting-related factors allowed the “clotting cascade” to be highlighted as enriched through Metascape analysis (Fig. S2), we investigated whether HFF-1 cells internalized serum factors. However, only peptides specific to human proteins were detected, and protein levels were either higher or lower under SBS compared to FBS exposure. While we cannot rule out such uptake, our data do not support this hypothesis. Changes in focal adhesion related to cell junction organization (Fig. S2) are found in the comparison between WBS and SBS, indicating that remodeling of ECM and cell-matrix interactions could also be impacted by changes in the serum composition between summer-active and hibernating bears. These effects could be mediated by some signaling pathways such as the MAPK or PI3K-AKT pathways, which are also among the enriched functions highlighted by the functional annotation enrichment analysis (Fig. 2C).

### Bear serum induces modulation of protein abundance related to extracellular matrix constituent, cell-matrix interactions, and immune system

**Fig. 3:**
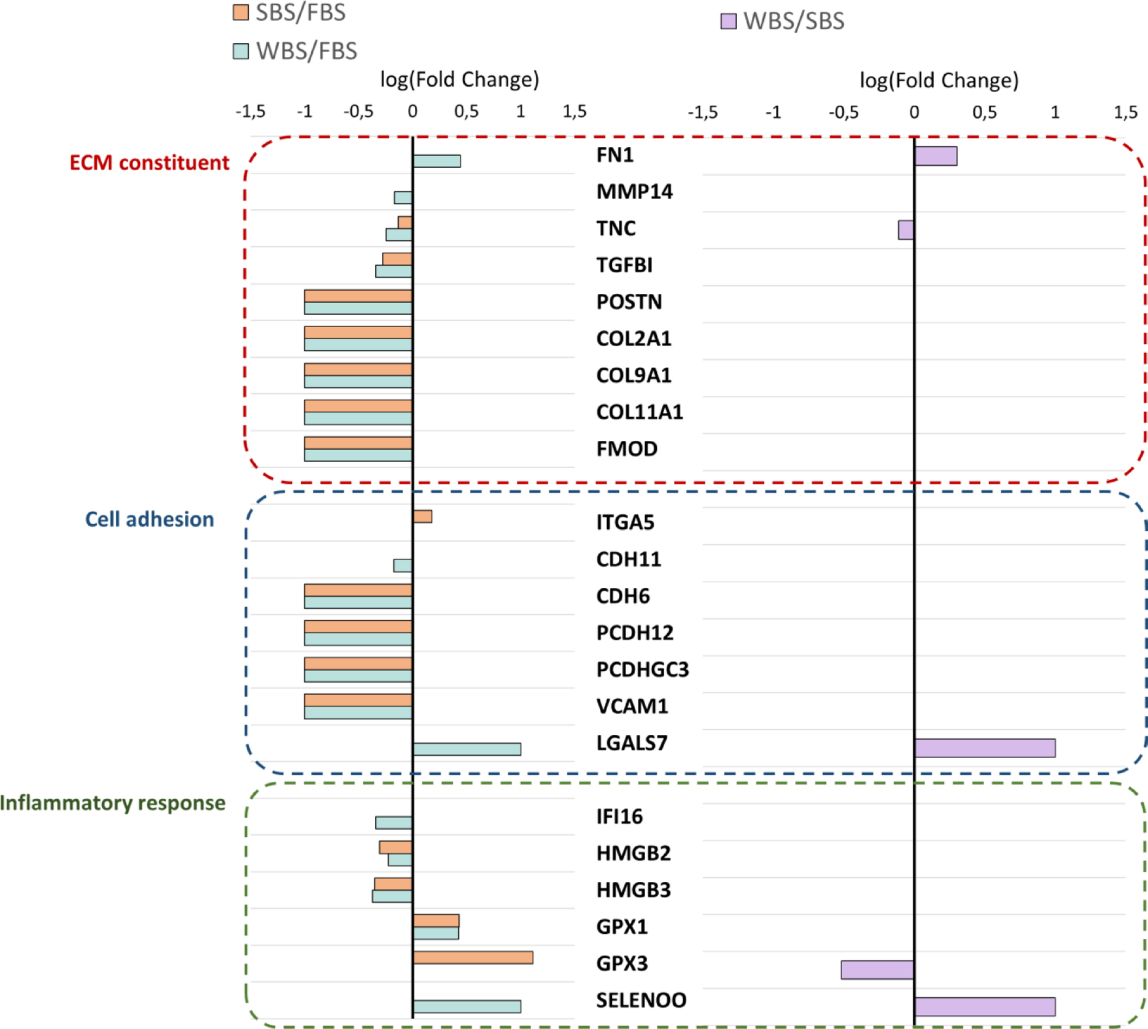
Fold changes in ECM, cell adhesion, and inflammatory proteins. Fold changes in the expression of proteins involved in extracellular matrix (ECM) organization, cell adhesion, and inflammatory response, based on proteomic analysis. The left panel shows the comparison between the bear serums conditions and the FBS control, while the right panel shows the comparison between hibernating and active bear serum. Only fold changes corresponding to statistically significant differences (*p* < 0.05, one-way ANOVA) are shown. For proteins undetected in one condition, an arbitrary value of 1 or − 1 was assigned to represent upregulated or downregulated proteins, respectively.

Since the regulation of extracellular matrix (ECM) structural components and cell adhesion factors was a key outcome of serum incubation, we next focused our attention on these processes. In cells treated with FBS, several matrix proteins were detected, including few collagens (COL2A1, COL11A1, COL9A1), periostin (POSTN) and fibromodulin (FMOD), while they were below the level of detection in bear serum (summer and winter)-treated cells. TGFBI (Transforming growth factor-beta-induced protein ig-h3), a cell adhesion molecule downstream of the TGF-β pathway, was also downregulated in SBS and WBS conditions compared to the FBS condition, suggesting that TGF-β signaling might be reduced by bear serum (Fig. 3). A stronger effect of WBS than SBS could be observed with downregulation of another matrix protein, Tenascin C (TNC). In contrast, upregulation of the matrix protein fibronectin (FN1) could be observed due to the WBS treatment compared with both the FBS and SBS conditions.

For cell adhesion proteins, which represent a key functional interface between the extracellular environment and intracellular structural organization and signaling pathways, downregulation of vascular cell adhesion molecule 1 (VCAM-1) was observed in cells treated with bear serums (summer and winter) compared with FBS, along with a concomitant decrease in cadherin and protocadherin receptor abundances (PCDH12, PCDHGC3, CDH6 and CDH11). Changes between the two bear conditions could be noted, with upregulation of LGALS7 (Galectin-7) only in WBS (Fig. 3), which is indicative of an alteration of cell-matrix interactions.

Given the tight interplay between tissue remodeling and immune activation^25^, we examined how the factors involved in immune response were regulated. Downregulation of immune related molecules such as IFI16 (Gamma-interferon-inducible protein 16), HMGB2 (High mobility group protein B2), and HMGB3 (High mobility group protein B3) in bear serum conditions compared with FBS condition was observed. In contrast, extracellular glutathione peroxidase 1 (GPX1) was markedly overexpressed in those same conditions, pointing toward an enhanced antioxidant response. However, the other antioxidant enzymes glutathione peroxidase 3 (GPX3) was downregulated only in WBS compared with SBS and Selenoprotein O (SELENOO) was upregulated in WBS compared with FBS and SBS. The bear serum, particularly WBS, therefore seems to regulate the redox balance in fibroblasts, concomitantly with a decrease in inflammatory immune related molecules.

Overall, the changes that were observed suggested alterations in intracellular signaling cascades. This led us to investigate MAPK and AKT signaling more precisely.

### Activation of MAPK (ERK) and AKT signaling in WBS condition

**Fig. 4:**
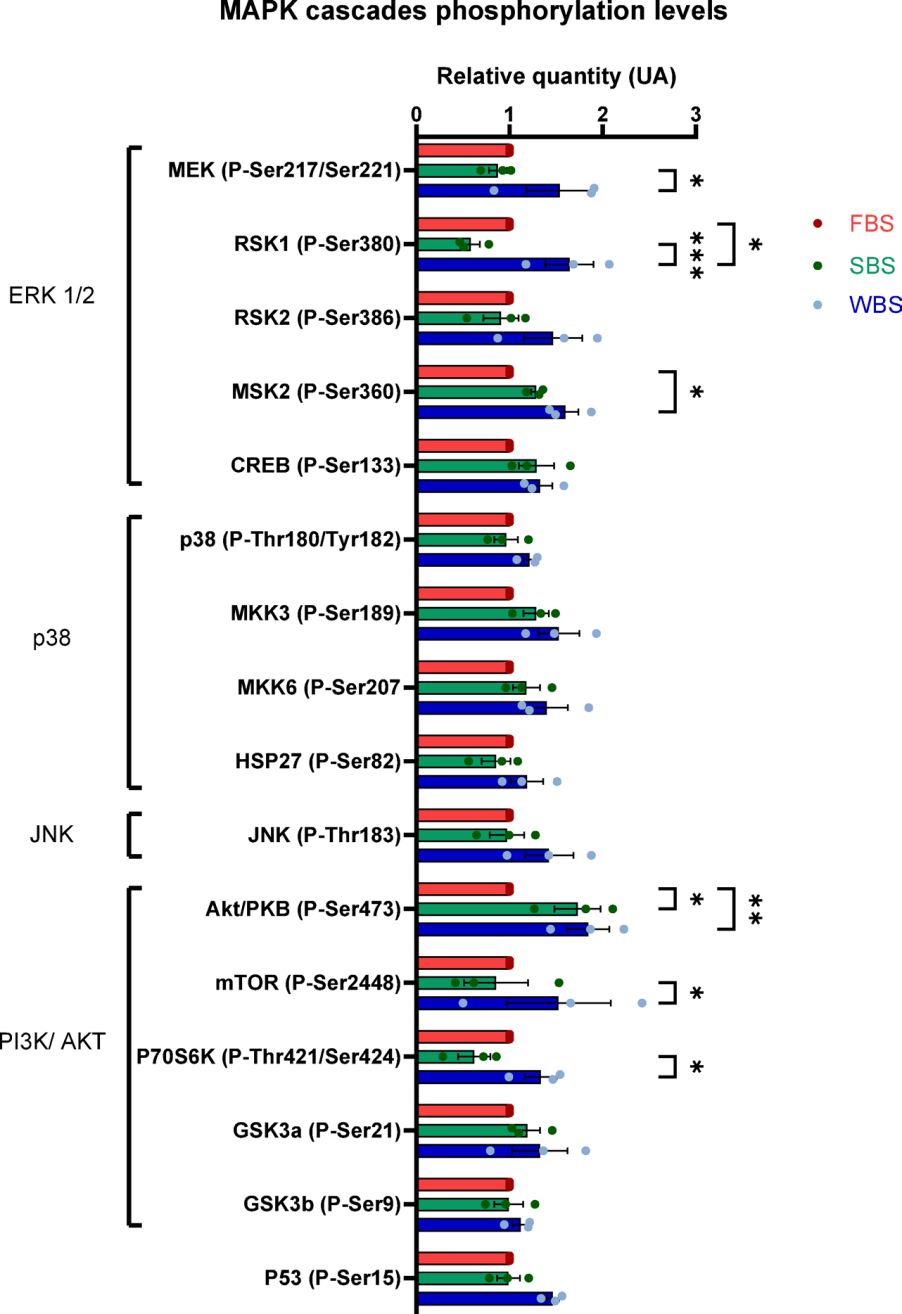
Relative activation of MAPK and AKT signaling assessed by protein phosphorylation. Phosphorylation levels of key proteins involved in the MAPK and AKT signaling pathways. UA is defined as the ratio of the mean value for FBS-, SBS- or WBS-treated samples to the mean value of the control (FBS) condition. Two-way ANOVA was performed to compare MAPK phosphorylation levels. Statistical significance is indicated by asterisks: *p < 0.05, **p < 0.01, ***p < 0.001.

A significant increase in the level of the phosphorylated forms of ERK downstream effectors, namely MEK1, MSK2, and RSK1, was observed in WBS condition compared with SBS and FBS, suggesting an activation of ERK signaling pathway by WBS (Fig. 4). Similarly, the AKT/mTOR/P70S6K pathway appeared to be activated, with a significant increase in the phosphorylated levels of AKT, mTOR, and P70S6K due to WBS compared with SBS (Fig. 4).

## Discussion

Hibernation is a state of metabolic suppression involving torpor, which enables certain animals to survive extreme environmental conditions of drastically reduced food availability and extreme environmental temperatures^26^. During hibernation, bears experience prolonged physical inactivity and food deprivation without any long-term adverse effects, whereas these conditions would be extremely harmful to humans^3^. Several studies have shown that bear serum can induce protective effects on various types of cells in vitro^6,27,15,10,11^. However, the underlying molecular mechanisms remain poorly understood. We hypothesized that these effects, mediated by factors circulating in the bear blood that are still to be identified, could be better understood by analyzing the proteome of fibroblast cells incubated with bear serum. Our study revealed a marked remodeling of the proteome of human fibroblasts by bear serum, indicative of the induction of an anti-fibrotic phenotype.

Since excessive deposition of ECM components is a hallmark of fibrosis^28^, down-regulation of ECM components, particularly collagen, suggests that bear serum, especially WBS, induces an anti-fibrotic effect in human fibroblasts. Moreover, the downregulation of TGFBI by bear serum (SBS and WBS) observed in our study suggests a repression of TGF-β pathway^29^. This finding is consistent with previous observations in human myotubes, where WBS inhibited the TGF-β, while maintaining the BMP pathway^9^. This shows that inhibition of the TGFβ pathway by bear serum can be induced in various types of cells. Inhibition of TGF-β signaling in human fibroblasts has been shown to exert anti-fibrotic effects by reducing collagen deposition^30^, whereas activation of TGF-β signaling in epithelial and fibroblastic cells leads to the accumulation of matrix components such as collagens^31^. Therefore, reduced accumulation of collagens after incubation of fibroblasts with bear serum, notably WBS, could likely be linked to TGF-β signaling inhibition. Importantly, TGFBI is not only a downstream target of the TGF-β pathway but also functions as an extracellular matrix protein that binds collagens and plays a role in cell adhesion^32^. Thus, its modulation by bear serum may impact both intracellular signaling and extracellular matrix organization, consistent with the anti-fibrotic signature observed. Accordingly, our hypothesis of TGF-β pathway inhibition in bear serum-treated cells compared to FBS-treated cells is further supported by regulation of few key adhesion factors. For example, VCAM-1 was not detected in fibroblast cells incubated with bear serum, and it is known that its expression is induced by TGF-β^33^. Furthermore, CDH11, which has been shown to control collagen production and TGF-β activation^34^, was downregulated in cells exposed to WBS compared to FBS, with no change observed in cells treated with SBS. This further supports the presence of circulating compounds in hibernating bear serum capable of inhibiting the TGF-β pathway and remodeling the ECM.

To go further, and because of the tight interplay between tissue remodeling and immune activation (Wynn and Vannella^35^), we examined the regulation of immune-related factors in cells exposed to the various types of serum. Inflammation is a well-established driver of fibrosis^28^. Therefore, the observed downregulation of immune-related molecules, such as IFI16, HMGB2, and HMGB3, upon exposure to bear serum suggests promising targets for more effective anti-fibrotic therapies. HMGB2 and HMGB3 have indeed been showed to contribute to fibrotic processes, and their downregulation has been associated with decreased fibrotic signaling^36,37^. HMGB3-mediated fibrosis is also specifically linked to TGF-β signaling^37^ This supports the hypothesis that the observed anti-fibrotic effects may, at least in part, result from TGF-β inhibition. In addition, IFI16 has been implicated in liver fibrosis, where its expression was positively correlated with key extracellular matrix (ECM) and fibrosis-related genes, such as COL1A1, COL3A1, and TGFB1. Furthermore in vitro knockdown of IFI16 induced anti-fibrotic effects, with a significant downregulation of these same collagens and TGF-β agonists^38,39^. The selective downregulation of IFI16 in WBS- compared to FBS-treated fibroblast cells therefore further reinforces the potential of hibernating bear serum to induce a pronounced anti-fibrotic pattern. Together, these findings support the hypothesis that hibernating bear serum can modulate cellular processes related to tissue remodeling and preservation, potentially through suppression of TGF-β–driven pathways, with a more pronounced effect observed under WBS treatment.

Along with these findings, differential modulation of antioxidant molecules was observed here between the conditions. Increased GPX1 expression was observed in both bear conditions (SBS and WBS) compared to FBS, indicating enhanced antioxidant capacity, consistent with previous observations in bear muscle and the general increase in tissue antioxidant defenses during hibernation^40,26^. In contrast, SELENOO was upregulated only in WBS-treated cells compared to SBS, while GPX3 expression was decreased, suggesting a distinct modulation of the antioxidant balance between the different serum types. Overall, the antioxidant properties of bear serum may thus help limit inflammation, therefore leading to additional protective and anti-fibrotic effects in fibroblasts.

Given that the observed changes suggest remodeling of the ECM as well as changes in cell-matrix and cell-cell interactions, we next focused our attention to key intracellular signaling pathways identified in the functional analysis of our proteomics data. In particular, we observed an upregulation of the phosphorylated proteins involved in the MAPK/ERK pathway in WBS-treated cells compared to the other conditions, suggesting activation of this signaling cascade. This potential pathway activation may further contribute to the anti-fibrotic effect of bear serum, especially the stronger effect observed with WBS. Indeed, previous findings have reported that activation of MAPK/ERK signaling and concomitant inhibition of TGF-β signaling can promote myofibroblast dedifferentiation, thereby counteracting the fibrotic phenotype^41^. In our model, an increase in GPX1 expression was observed under bear serum treatment. It has been shown that GPX1 overexpression activates the AKT pathway in non-small cell lung cancer^42^. Consistently, we detected increased levels of phosphorylated AKT with both SBS and WBS, compared to FBS. Increased phosphorylated levels of mTOR and P70S6K were only observed in WBS vs. FBS. These findings confirm the activation of the PI3K/AKT signaling pathway, especially in WBS condition. This pathway plays a complex, context-dependent role in fibrosis, exhibiting both pro- and anti-fibrotic effects. Simultaneous activation of AKT pathway and inhibition of the canonical TGF-β1/Smads cascade have been associated with anti-fibrotic effects in liver fibrosis^43,44^. However, other studies on pulmonary and cystic fibrosis have reported that AKT activation can promote fibrosis by supporting cell survival, proliferation, migration and ECM production^45,46^, indicating the effects of this pathway are context-dependent. In our model, AKT pathway activation appears to be associated with anti-fibrotic effects. It could be possible that the PI3K/AKT signaling represents an additional factor regulated by bear serum to exert an anti-fibrotic effect or to help finely modulate this effect, likely in coordination with other pathways, including TGF-β1 inhibition.

Overall, most of the differences were observed between FBS-treated cells and bear serum-treated cells. This outcome was expected since FBS, being of fetal origin, has a markedly different composition from subadult serum, particularly regarding growth factors and protein content. This could also be attributed to the species from which the serum originates. However, despite that, differences between the effects of SBS and WBS were observed, with more pronounced effects seen with serum from hibernating bears, particularly with regard to the induction of an anti-fibrosis phenotype. Our in vitro observations are also corroborated by the anti-fibrotic phenotype exhibited in vivo by 13-lined ground squirrels during hibernation^47^. Interestingly, this was confirmed in vivo by a transcriptomic approach on bear muscles biopsy where a down regulation of gene involved in ECM organization effect has also been observed^48^. This could reflect a tissue protection mechanism during hibernation. Attenuation of fibrotic processes is widely recognized as a protective condition, as excessive fibrosis leads to pathological tissue remodeling, impaired regeneration and progressive loss of function. Limiting or delaying fibrosis is therefore a valid strategy to maintaining tissue integrity and promoting recovery after various types of stress or injury^49^. Thus, replicating protective conditions similar to hibernation in human cells by incubating them with serum from hibernating bears holds great promise for better understanding these protective effects, which likely involve multiple signaling pathways as discussed above. Identifying the serum components that are capable of inducing this protective phenotype would help open up new avenues for the development of innovative therapies to treat fibrosis in humans. Moreover, determining whether the differential effect between winter and summer bear serum is due to the presence of activators and/or inhibitors within the bear serum remains to be further explored.

## Supplementary Information

Below is the link to the electronic supplementary material.

Supplementary Material 1

Supplementary Material 2

## Data Availability

All data used in this study are available in the main text or in supplementary materials. The mass spectrometry proteomics data have been deposited to the ProteomeXchange Consortium via the PRIDE (Vizcaíno et al., 2016) partner repository with the dataset identifier PXD069199.
