# Supplementary material for: Hibernating bear serum triggers an anti-fibrotic signature in human fibroblasts, involving ECM remodeling and MAPK signaling activation
Source: Sci Rep. 2026 Mar 21;16:14434. doi: 10.1038/s41598-026-43734-y (PMC13150032; doi:10.1038/s41598-026-43734-y)
Supplement: Supplementary file 2 — Supplementary Material 2 [file 41598_2026_43734_MOESM2_ESM.pdf]

Hibernating Bear Serum Triggers an Anti-fibrotic Signature in Human Fibroblasts, involving ECM Remodeling and MAPK Signaling Activation

Jade Sutter, Alexandre Geffroy, Amandine Moretton, Anne Randi Græsli, Jonas Kindberg, Lydie Combaret, Etienne Lefai, Isabelle Garreau-Balandier, Fabrice Bertile, Patrick Vernet

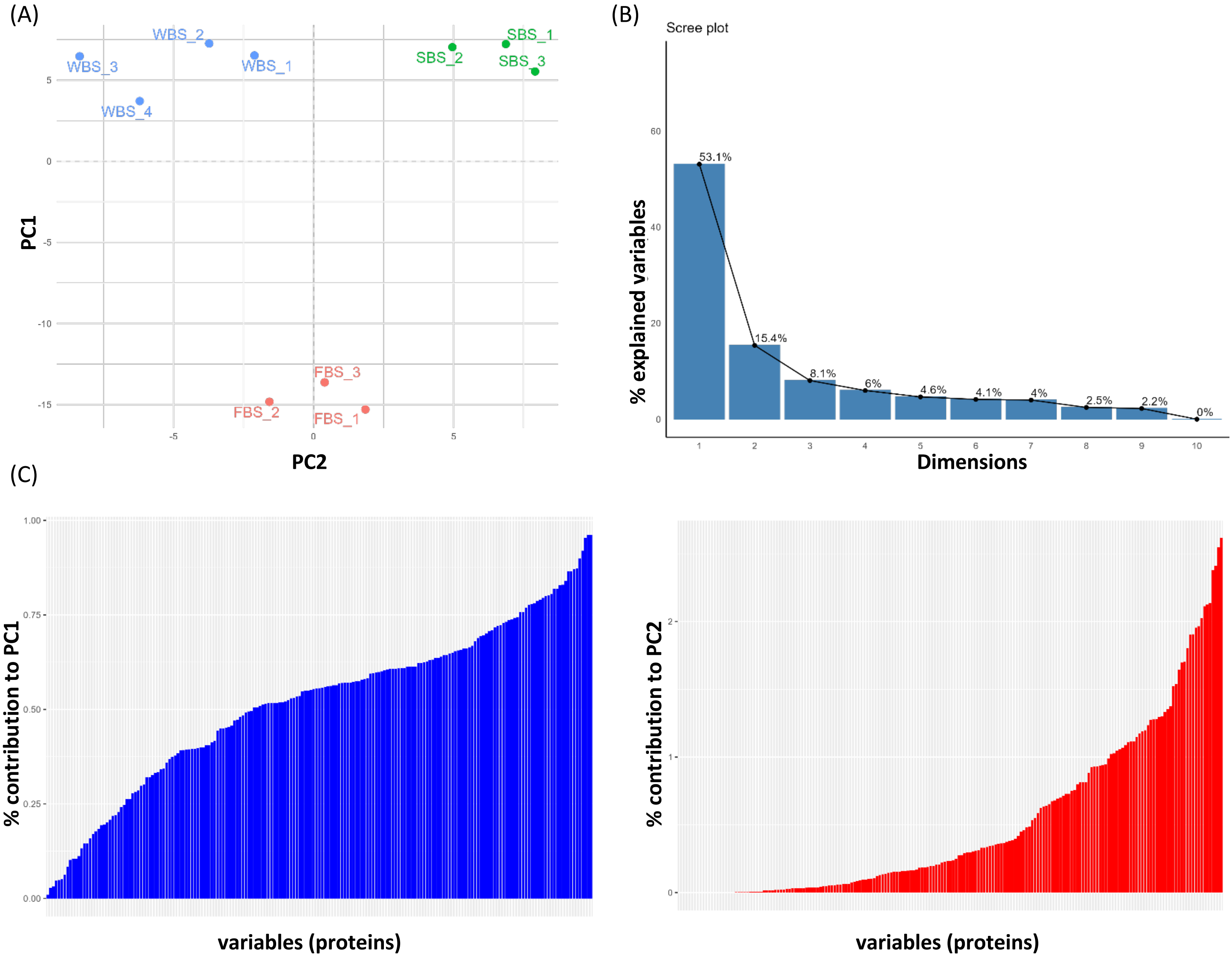

Supplementary Figure S1: Principal component analysis (PCA) of differentially expressed proteins. (A) PCA performed using the relative abundance of differentially expressed proteins across all samples, highlighting that samples from different experimental conditions were nicely discriminated. (B) Bar plot showing the percentage of variance explained by each principal component. (C) Contribution of individual proteins to principal component 1 (PC1) and principal component 2 (PC2), indicating the main drivers of variance in the dataset.

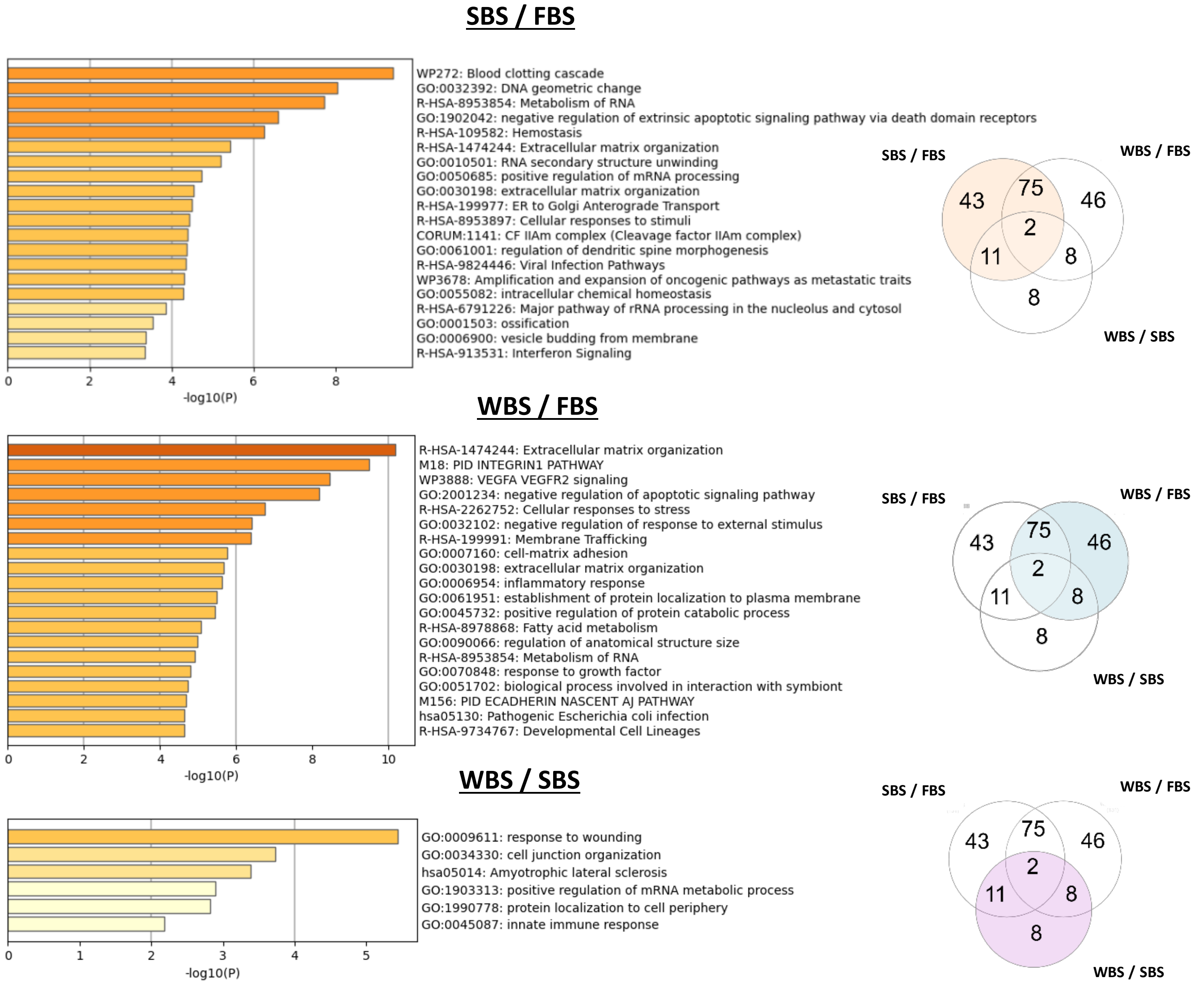

Supplementary Figure S2: Functional annotation enrichment. Bar plots showing the most enriched Gene Ontology terms as identified by Metascape, ranked by p-value, with Venn diagrams illustrating number of differentially expressed proteins that overlap in pairwise comparisons of different conditions.
